# Supplementary material for: Gender differences in dry eye disease symptoms associated with psychological health indicators among adults using mobile mental health apps
Source: PLoS One. 2023 Jan 17;18(1):e0278921. doi: 10.1371/journal.pone.0278921 (PMC9844882; doi:10.1371/journal.pone.0278921)
Supplement: Attachment — Submitted filename: comments.docx [file pone.0278921.s002.docx]

In Result part:

1. Line 94-95: The first sentence was wrong. Please remove.

2. It would be better result if the author can analyses more in each age because as we know that dry eye mostly happens in aging female.
