# Supplementary material for: Gender differences in dry eye disease symptoms associated with psychological health indicators among adults using mobile mental health apps
Source: PLoS One. 2023 Jan 17;18(1):e0278921. doi: 10.1371/journal.pone.0278921 (PMC9844882; doi:10.1371/journal.pone.0278921)
Supplement: Attachment — Submitted filename: Response to Reviewers_2.doc [file pone.0278921.s003.doc]

Sep 20, 2022

Keisuke Suzuki, MD, PhD
Editorial Board Member
*Plos One*

Dear Dr. Susuki:

We would like to thank you and the reviewers of the *Plos One* for taking the time to review our article. We revised the manuscript, based on your comments. The changes are summarized below:

**Reviewer #1:**

**Comment**

The manuscript entitled “Gender Differences in Dry Eye Disease symptoms associated with Psychological Health indicators among Adults using mobile mental health apps” was an interesting research. The following comments can help the authors to improve it:

1-Please follow the journal instruction to provide a structured abstract if possible. **[Response] Thank you for your comments. We revised it to be a structured abstract.**

2-The whole manuscript needs to be revised in terms of English language.

**[Response] Thank you for your comments.** The manuscript was edited by English speaker.

3-The reference numbers in the main text are not sequential, and need to be corrected. **[Response] Thank you for your comments. We confirmed the reference numbers again. Also, 6 references (number of 9, 10, 11, 12, 16, 17) have been added.**

4-Abbreviations need to be presented in their full form in the first place that they are used.

**[Response] Thank you for your comments. We have corrected the abbreviations to the submission format.**

5-The introduction section is so brief and the gap of knowledge needs to be explained with more details.

**[Response] Thank you for your comments.** **We added the introduction section. (Page 4, lines 61, 66-71)**

6- More information about the App needs to be added to the manuscript.

**[Response] Thank you for your comments. We added the App information in method section. (Page 4, lines 87-89)**

7. More information about the settings of the study and the reasons for excluding patients aged 61 or more need to be added.

**[Response] We agree with your comments. We added the reasons for excluding patients aged 61 or more and references. (Page 5, lines 95-98)**

**The previous study has demonstrated that over 60 age was a significant risk factor for dry eye symptoms. Therefore, older participants over 60 were not included. Also, we revised the manuscript to be exactly 60 years old. In addition, because it was reported that those over the age of 60 who used health apps had low self-efficacy, we excluded those over the age of 60.**

8-As the number of women was higher than men, is there any possibility for bias in reporting the results? Why didn’t the researchers recruit the same number of patients for males and females?

**[Response] Thank you for your comments.**

**There were more women who participated to use the health app. Therefore, we did not adjust to match the number of men and women in our statistical analysis. Since we showed the analysis results for men and women separately in Tables 1, 2, and 3, we think that it would not affected the results.**

**Reviewer #2:**

**Comment**

In Result part:
1. Line 94-95: The first sentence was wrong. Please remove.

**[Response] Thank you for your comments. We removed the sentence.**

2. It would be better result if the author can analyses more in each age because as we know that dry eye mostly happens in aging female.

**[Response] Many thanks for your comments.**

**We considered dividing the analysis into each age groups, but we did not include such an analysis because we excluded the old female group by adding to the reasons for excluding the age of 60 or older in the method. In addition, these sentences were described to the Discussion section. (page 16, line 303)**

Sincerely yours,
