# Supplementary material for: Gender differences in dry eye disease symptoms associated with psychological health indicators among adults using mobile mental health apps
Source: PLoS One. 2023 Jan 17;18(1):e0278921. doi: 10.1371/journal.pone.0278921 (PMC9844882; doi:10.1371/journal.pone.0278921)
Supplement: Attachment — Submitted filename: Review comments_1023_1.docx [file pone.0278921.s004.docx]

Reviewer #1: I appreciate the authors for their time and efforts to revise the manuscript. However, it still needs to be improved in the following areas:

1- Again, the whole manuscript needs to be revised in terms of English language. Moreover, please be consistent when using (.), (-) and (,).

→Answer: We have revised all the marks to be consistent throughout the paper. Also, the manuscript was edited by an English native speaker.

2- To report the results, we unusually use “sex” which is categorized to “male” and “female”. Please consider these two in the whole manuscript.

→Answer: We have revised all “men / women” to “male / female” throughout the paper.

3- In the introduction section, the terms sex and gender were used next to each other. Please only use one term "sex".

→Answer: We have revised all “gender” to “sex” throughout the paper.

4- Please revise the first paragraph of the methods section, if you are explaining inclusion and exclusion criteria in other paragraphs.

→Answer: We have consolidated the paragraphs and modified them to fit the context.

5- More information about the App needs to be added to the manuscript. Adding only two lines to the methods section is not adequate. Please add some figures of the App, too.

→Answer: We have added some figures for improvement.

6- More information about the settings of the study needs to be added.
→Answer: As a primary study, this study was first aimed at examining the participants' mental health and dry eye symptoms. In the future, we plan to have participants use the healing program on a regular basis through a mobile app to investigate the intervention effect.

7- In the tables, there are a number of cells which show no label or figure. Please complete the tables or merge cells, etc.

→Answer: We follow the reviewer’s suggestion.
